# Emotion knowledge, social behaviour and locomotor activity predict the mathematic performance in 706 preschool children

**DOI:** 10.1038/s41598-021-93706-7

**Published:** 2021-07-13

**Authors:** Thalia Cavadini, Sylvie Richard, Nathalie Dalla-Libera, Edouard Gentaz

**Affiliations:** 1 Department of Psychology, University of Geneva, Geneva, Switzerland; 2 Valais University of Teacher Education, Saint-Maurice, Switzerland; 3 Savoie Department, Minister of National Education, Paris, France; 4 Swiss Center for Affective Sciences, University of Geneva, Geneva, Switzerland; 5 CNRS, Grenoble, France

**Keywords:** Neuroscience, Psychology

## Abstract

What are the foundational abilities that young children must develop at the beginning of school for their future academic success? Little is known about how emotion knowledge, social behaviour, and locomotor activity are associated and how these abilities may be predictors of academic-mathematic performance (less correlated with the children’s SES than pre-reading and linguistic achievement) in a large cohort of preschool children. Here we show that emotion knowledge, locomotor activity, social behaviour, and academic-mathematic performance are interrelated in 706 French preschool children aged 3 to 6. Mediation analyses reveal that the increase in academic-mathematic performance is explained by the increases in emotion knowledge and social behaviour and, in turn, children with a greater comprehension of emotions tend to have better locomotor skills and higher academic-mathematic scores. Additionally, sequential mediation analysis reveals that the increase in emotion knowledge, locomotor activity and social behaviour partially explains the increase in academic-mathematic performance. These results are discussed in relation to three possible mechanisms. Our findings are consistent with the political and scientific consensus on the importance of social-emotional abilities in the academic world at the beginning of school and suggest adding locomotor activity to these foundational abilities.

## Introduction

An increased number of studies have examined the foundational abilities that prepare children for school and are particularly essential for progress in the academic world. Indeed, it is during this period at the beginning of school that children develop these abilities for their future academic success. Among these abilities, “emotion knowledge” significantly contributes. The term *emotion knowledge* is often used to describe the capacity to understand emotion in facial expressions, behavioural cues, and social contexts^1^. In young children emotion knowledge is generally defined as the ability to recognize emotions, label emotional facial expressions, and identify situations that generate emotions^2,3^. Several studies have demonstrated that emotion knowledge was linked to social competence and academic achievement in young children^4–6^. A recent meta-analysis of studies with children (aged 3 to 12) revealed that higher level in emotion knowledge tend to be more successful in the academic performance, peer acceptance, and in school adjustment^7^. More particularly, preschool emotion knowledge was a significant predictor of academic achievement at first grade assessed by “The Letter-Word” identification subtest, by “The Dictation” subtest and by a subtest measuring the children’s ability to analyse and solve practical math problems^8^. Emotion knowledge was positively correlated with school achievement including concept knowledge (e.g., naming and identifying colours, identifying concepts such as "small" vs "large", etc.) and language competence (e.g., the ability to use age-appropriate nouns and verbs, etc.) in preschoolers^9^.

Interestingly, in a recent meta-analysis, MacCann et al. (2020) examined the relation between student emotional intelligence and academic performance observed from elementary school to university. They found an overall effect of ρ = 0.20 using robust variance estimation and proposed three underlining mechanisms: (1) building social relationships at school (higher emotional intelligence may be linked to a better management of the social world, relationships with teachers, peers, and family); (2) regulating academic emotions (higher emotional intelligence may be linked to better regulations of negative emotions) and (3) academic content overlap with emotional intelligence^10^. The recognition of primary facial emotion expressions (e.g., joy, sadness, anger, fear) is very important for the development of emotion understanding and adapted social interaction^11^. According to Izard et al. (2001), the ability of recognizing and labelling emotion expressions in 72 children at age 5 is a long-term predictor of their social behaviour and academic performance at age 9^5^. Therefore, the positive social interactions were facilitated by the ability level of detecting and labelling emotions.

Moreover, some studies highlighted the mediating role played by social competence on academic performance. A longitudinal study following 73 children at ages 5, 7, and 8, showed that social understanding at age 5 predicted social competence at age 7, which in turn predicted school achievement at age 8. Results also showed that the social competence mediated the association between early social understanding (including belief understanding and emotion understanding) and later school achievement^12^.

In this vein, the quality of the relationships with teachers and peers at the beginning of the preschool year predicted increases in emotion knowledge during the preschool year and kindergarten academic achievement assessed by emergent literacy and early numeracy skills. However, complementary analyses revealed that emotion knowledge mediated the link between interpersonal relationships and academic achievement^13^. Together, these results suggest that emotion knowledge predict socio-behavioural development and academic performance in young children.

Locomotor activity, especially in the context of play, is an integral part of young children's everyday life and could also affect the development of emotion understanding abilities, social behaviour, and consequently academic performance. Research shows that young children need to engage in physical activity for psychological development. More particularly, gross locomotor movements in the context of play increase from the toddler to preschool period and then decline during primary school, with a likely peak around 4 to 5 years^14^. Children aged 5 to 9 years were more attentive after the break spent outside suggesting that locomotor activity seems consequently to influence some cognitive abilities^15^. A longitudinal study showed a significant predictive relation between early gross motor development and later school aged cognitive development, particularly working memory and processing speed^16^. Moreover, some studies suggest that motor skills may influence academic achievement. Early kindergarten motor skills predicted achievement in reading and mathematics at the end of first grade^17^. Children aged 7 to 12 with learning disabilities exhibited poorer scores in gross motor skills. Especially, a specific relation between reading and locomotor skills (e.g., run, gallop, hop, leap, jump) was found, and a trend was shown between mathematics and object-control skills (e.g., two-hand strike, stationary bounce, catch, kick) in children with learning disabilities^18^. More specifically related to basic numerical abilities in kindergarteners, fine and gross motor skills accounted for variance respectively in nonsymbolic number line estimation and in nonsymbolic magnitude comparison^19^. In this recent study, the researchers indicated that the contribution of motor skills to the development of basic numerical skills is widely unexplored, which is one of the reasons why we focused specifically on mathematic skills. In addition to having an impact on cognitive abilities and academic achievement, locomotor activity seems to promote emotion regulation in young children. Indeed, physical play predicted emotion regulation one year later in 122 preschoolers, suggesting that physical play could allow children to practice expressing and controlling their emotions^20^. Locomotor activity in preschoolers would therefore be closely linked to their emotional development. A review of studies published between 1997 and 2007 show that children who exhibited emotion, behavioural, and pervasive developmental disorders presented poor gross motor performance and problematic self-perception of motor competence^21^. As suggested by the authors, these motor impairments of children with emotional, behavioural, and pervasive developmental disorders may lead to low self-perceived motor competence, which in turn may have a negative effect on their participation in activities that require movement, such as play and sports with peers, and consequently impedes psychosocial development and experiences that support the improvement of their motor performance. Which leads us to think that motor skills would contribute to positive peer relationships in the early years. As mentioned above, there is likely a circular relation between poor motor skills and emotional difficulties. Children with emotional problems may be more hesitant and less able to play on the playground with their peers, which may reinforce these emotional problems^22^ and as a result the development of locomotor skills. Indeed, kindergartener children with low motor abilities were less likely to engage in social play and exhibited higher frequency of social reticence during free play^23^. In sum, locomotor development in young children may therefore predict cognitive, social-emotional abilities, and academic development.

In the present study, participants (*N* = 706) aged 3 to 6 completed assessments of their emotion knowledge, social behaviour, and locomotor activity as well as their academic-mathematic performance (measured by three mathematic-numerical sub-tasks) in order to better understand the relations between the different measures and the academic performance in mathematics (computed from the results of the three sub-tasks) and how these three abilities may be predictive of academic-mathematic performance of kindergarten pupils before they enter elementary school. We chose only to assess academic achievement with mathematics-numerical performances for three main reasons: (1) as above mentioned, the contribution of motor skills to the development of basic numerical abilities is widely unexplored in the literature, (2) mathematic performances were correlated, but lower than reading skills, with the French-speaking children’s socio-economic status (SES)^24,25^, and (3) the assessments made by the teachers were already numerous and it was not possible to adding an additional measure. We hypothesized (1) that these abilities (emotion knowledge, social behaviour, locomotor activity, and academic-mathematic performance) should be associated with each other and with the age of the children, just as they should also increase with age, (2) that emotion knowledge, social behaviour, and locomotor activity, in addition to age, should be predictors of academic-mathematic performance. Furthermore, considering previous studies that have already investigated some potential associations between these variables^4,12,13,26^, we performed mediation analyses to test our last hypothesis that (3a) social behaviour mediates the relation between emotion knowledge and academic-mathematic performance on the one hand, and (3b) emotion knowledge mediates the relation between locomotor activity and academic-mathematic performance on the other hand.

The assessment was conducted in close collaboration with 33 experienced preschool teachers, who took part in an interactive workshop in which they were trained to set up, perform, and rate, in standardized ways, the emotion task. The emotion task is composed of three sub-tasks aimed (1) at recognizing the primary emotions of anger, fear, joy, sadness, and neutral expression, and (2) at understanding their causes by (a) pointing out, and (b) naming these same emotions when they are felt by others in given situations. We adapted the emotion task from previous work^27,28^ to measure young children’s emotion knowledge (see Supplementary Material to this manuscript). After this initial workshop, the teachers were continuously monitored via a digital platform through which we followed the task's implementation in the classrooms as well. The evaluations of academic-mathematic performance, social behaviour, and locomotor activity (see “Methods” section) were chosen in close collaboration with teachers and were adapted according to the “official teaching aids” of the French educational system (a schematic representation of the official learning domains in French preschools, highlighting the competencies of interest in the present study, is available in Supplementary Fig. S1). Results for the latter three measures were calculated as percentages of success, whereas the total emotion knowledge score ranged from 0 to 15 (1 point per correct answer to each of the three sub-tasks). Considering that pupils were nested within different classrooms and that 33 teachers conducted the assessments, we investigated the critical assumption regarding independence of observations before conducting the statistical analyses. For this purpose, we calculated an intraclass correlation coefficient (ICC) with a fully unconditional model (i.e., considering the assessing teacher's effect random) using the mixed model procedure in SPSS. An ICC of 9.69% was thus obtained, which was not trivial (i.e., greater than 10% of the total variance in the mathematic outcome)^29^. We therefore assumed that it was reasonable to consider that there was no violation of the independence assumption, and consequently that it was not required to account for the hierarchical structure of our data using multilevel methods.

**Figure 1:**
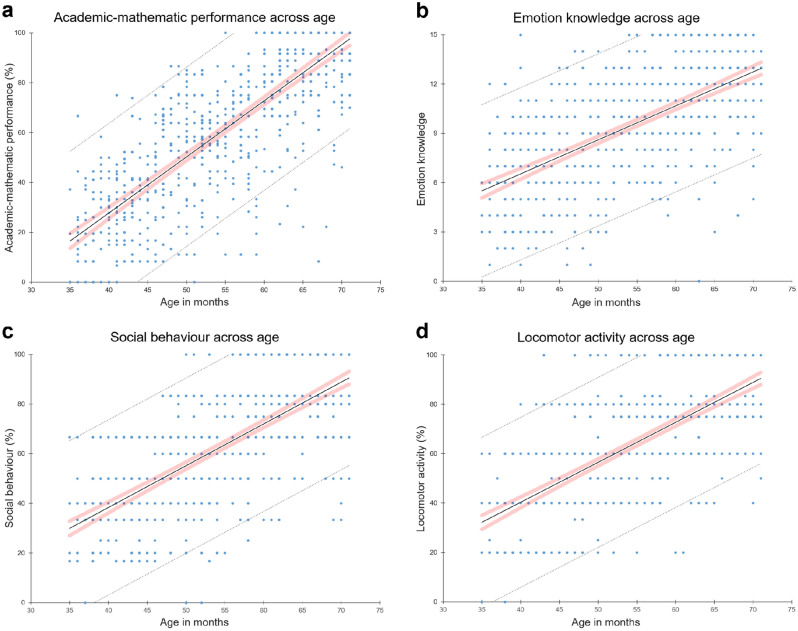
Scatterplots of observed values across age for all variables. (**a**–**d**) Differences in (**a**) academic-mathematic performance^1^, (**b**) emotion knowledge^2^, (**c**) social behaviour^1^, and (**d**) locomotor activity^1^ all tended to correlate with age although graphs c and d seemed to fit less than the others (mathematics and emotion knowledge), which may be partly due to the fact that the social behaviour and locomotor activity assessment tasks were both more age-adjustable. Lines of best fit, 95% confidence intervals (red shading), and prediction intervals (grey dashed lines) were obtained from simple linear regression analyses. *Note*. ^1**.**^scores expressed as percentages of success; ^2**.**^scores ranged from 0 to 15 (1 point per correct answer).

Then, we first explored the interconnections between our/these variables by performing partial correlations controlling for gender, living location (rural, village, urban areas), and assessing teacher independent variables. As hypothesized, the correlation matrix thus generated (see partial Pearson’s *r* presented in Table 1) revealed that academic-mathematic performance scores correlated significantly with emotion knowledge (*r* = 0.618) as well as with social behaviour (*r* = 0.687) and locomotor activity (*r* = 0.660). In addition, the observed correlations between each of these three hypothesized predictors were also significant: emotion knowledge was positively correlated with social behaviour (*r* = 0.499) and with locomotor activity (*r* = 0.513), which also correlated positively with social behaviour (*r* = 0.714). Figure 1 illustrates these associations, showing the simple linear regressions conducted with age in months as the continuous regressor, which revealed that scores in academic-mathematic performance (standardized beta (*β*) = 0.789, *p* < 0.001), emotion knowledge (*β* = 0.629, *p* < 0.001), social behaviour (*β* = 0.699, *p* < 0.001), and locomotor activity (*β* = 0.694, *p* < 0.001) all increased with age, even though the regression results for social behaviour and locomotor activity (graphs c and d in Fig. 1) were more spread around the fitted line than the others. This may be partly explained by the fact that the tasks used to measure these variables (cooperative games and agility trail, both assessed by the teachers using observation grids based on the official teaching aids we provided them and then blind-coded by two experimenters, cf. “Methods” section) could be more age- or grade-adjusted than the emotion task, which was strictly the same for all participants, whereas the academic-mathematic performance scores, that fitted best, were more robust as they were computed from the results of three mathematic-numerical sub-tasks.

**Table 1:** Partial correlations (Pearson’s* r*), means, and standard deviations for all variables (*N* = 706) controlling for the following independent variables: gender, living location, and assessing teacher.

	1	2	3	4	*M*	*SD*
1. Age in months					54.354	10.412
2. Academic-mathematic performance^a^	.788*				60.106	29.700
3. Emotion knowledge^b^	.633*	.618*			9.507	3.424
4. Social behaviour^a^	.703*	.687*	.499*		62.554	25.081
5. Locomotor activity^a^	.697*	.660*	.513*	.714*	63.532	24.289

*Note*. Marked correlations (*) are significant at *p* < .001.

^a^Score expressed as a percentage of success.

^b^Total score range: 0 to 15 (1 point per correct answer).

We then specifically focused on mathematics performance and investigated whether it could be better predicted by considering other variables than the age factor alone. Therefore, we ran hierarchical regression analyses to examine the weight of our hypothesized predictors (i.e., age, emotion knowledge, social behaviour, and locomotor activity). Furthermore, given the strong partial intercorrelations previously observed between these variables and children's age when controlling for their gender, living location and assessing teacher, we decided to also take these three independent variables into account in our analyses. Thus, a total of seven potential predictors were entered into the stepwise regression procedure in order to determine the best predictive model accounting for differences in academic-mathematic performance. The final hierarchical model (i.e., Model 4) so generated retained only four significantly predictive variables (cf. Table 2): age (standardized beta (*β*) = 0.469, *t*_(701)_ = 13.112, *p* < 0.001, Cohen’s *f*^2^ = 0.085), social behaviour (*β* = 0.213, *t*_(701)_ = 6.569, *p* < 0.001, *f*^2^ = 0.02), emotion knowledge (*β* = 0.169, *t*_(701)_ = , *p* < 0.001, *f*^2^ = 0.017), and locomotor activity (*β* = 0.094, *t*_(701)_ = 2.902, *p* = 0.004, *f*^2^ = 0.004) accounting for 68.1% of the variance in academic-mathematic performance (*F*_(4, 701)_ for R^2^ change = 8.421, *p* = 0.004).

**Table 2:** Final hierarchical regression model retained by the stepwise procedure predicting academic-mathematic performance. A total of seven predictor variables were entered into the analysis (gender, living location, assessing teacher, age, social behaviour, emotion knowledge, and locomotor activity) but only these last four were included in Model 4.

	Academic-mathematic performance			
	Standardized beta (*β*)	*t*_(701)_	ΔR^2^	R^2^
**Model 4**				0.681
Age in months	0.469	13.112**	0.622**	
Social behaviour	0.213	6.569**	0.036**	
Emotion knowledge	0.169	6.121**	0.019**	
Locomotor activity	0.094	2.902*	0.004*	
	*F*_(4, 701)_ for *R*^2^ change = 8.421, *p* = .004			

*Note*. Durbin-Watson test = 1.687. As this value falls between 1 and 3, we are able to consider that the hierarchical regression’s condition concerning the independence of the errors is satisfied.

*p < .05, **p < .001.

**Figure 2:**
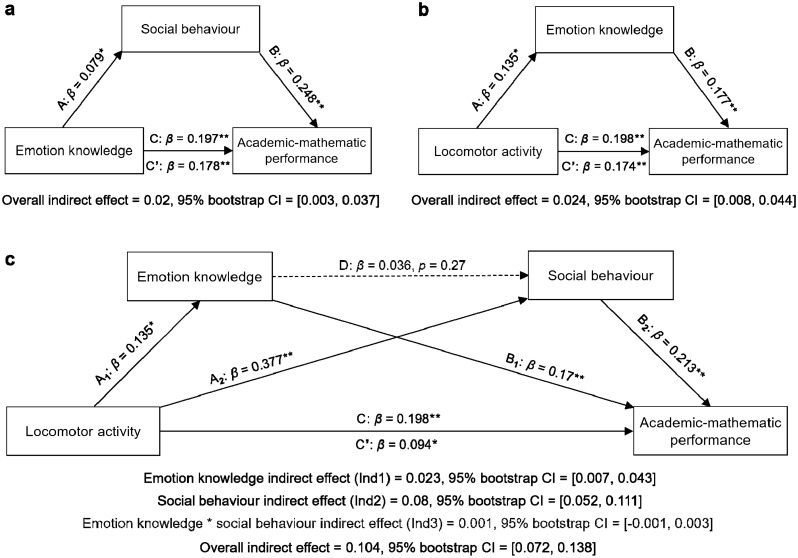
Results of mediation analyses controlling for the continuous variable age, entered as covariate (standardized coefficients and indirect effects are reported). **a**,**b** Simple mediation models suggested that (**a**) social behaviour mediated the effect of emotion knowledge on academic-mathematic performance, and (**b**) emotion knowledge mediated the effect of locomotor activity on academic-mathematic performance. (**c**) An additional sequential mediation model with two mediators highlighted that the effect of locomotor activity (X) on academic-mathematic performance (Y) was mediated by the indirect effects of both emotion knowledge (M1) and social behaviour (M2), whereas neither the indirect effect reflecting the sequential mediation (path A_1_DB_2_; effect of X through M1 to M2 and then to Y), nor the relation between emotion knowledge and social behaviour (path D, where M1 was serving as a predictor of M2) were significant. Standardized coefficients (*β*) and percentile bootstrap confidence intervals (CI) of 95% for all mediation models were obtained using 5′000 bootstrap resamples. Capital letters denote statistics for mediation pathways. *p < .05, **p < .001.

To test our mediation hypotheses, we conducted a robust bootstrapped mediation with 5′000 resamples controlling only for the variable age (entered as covariate) since neither gender, living location, nor assessing teacher were found to be significant predictors. This highlighted that (a) differences in social behaviour significantly mediated higher academic-mathematic performance with greater emotion knowledge since the completely standardized indirect effect of 0.02 (path AB in Fig. 2a) was statistically significant (95% bootstrap CI = [0.003, 0.037]), just as (b) emotion knowledge significantly mediated the effect of locomotor activity on academic-mathematic scores: the indirect effect of 0.024 was also definitively different from zero (path AB in Fig. 2b), as the bootstrap CI did not included zero neither (95% bootstrap CI = [0.008, 0.044]), indicating that children with greater comprehension of emotions tended to perform better in locomotor activities along with higher academic-mathematic scores. Although the direct effect of X on Y (path C’) was smaller than the total effect (path C) in each of the two simple mediation models performed, it still remained significant in both cases despite the influence of the mediator (see Fig. 2a,b).

Since the previous results showed that locomotor activity could predict emotion knowledge, and emotion knowledge could predict social behaviour, we then became interested in testing whether the two simple mediation models we conducted could merge into a multiple mediation model. For this purpose, we performed a sequential mediation analysis with two mediators (M1, emotion knowledge; M2, social behaviour), still controlling for the age covariate. In such a model, the effect of locomotor activity on academic-mathematic performance could be examined being meditated three ways: by paths A_1_B_1_ and A_2_B_2_ (Fig. 2c), through which it was mediated by M1 and M2 respectively, and by path A_1_DB_2_ which captured the sequential mediation (i.e., effect of X going through M1 to M2 and then to Y).

The sequential mediation analysis revealed that the indirect effect of emotion knowledge (completely standardized Ind1 = 0.023) was significant as its confidence interval was entirely above zero (95% bootstrap CI = [0.007, 0.043]), indicating that the relation between locomotor activity and academic-mathematic performance was mediated by emotion knowledge. Similarly, the indirect effect of social behaviour (Ind2 = 0.08) was also significant since its confidence interval did not include zero either (95% bootstrap CI = [0.052, 0.111]). This suggested that social behaviour also exerted an effect on the relation between locomotor activity and academic-mathematic performance. In contrast, the third indirect effect (Ind3 = 0.001; computed as the product of pathways A_1_, D and B_2_) was not significant because zero fell between the lower and upper bounds of its confidence interval (95% bootstrap CI = [− 0.001, 0.003]). Therefore, the sequential mediation captured by Ind3 was not significant, which can be explained by the fact that path D—where emotion knowledge served as a predictor of social behaviour—was not significant either (standardized beta (*β*) = 0.036, *p* = 0.27).

Although this sequential mediation model had a fixed direction, sequential mediation remained non-significant when the order of emotion knowledge and social behaviour were reversed (95% bootstrap CI = [− 0.003, 0.009]). However, the model’s overall indirect effect of 0.104 (computed as the sum of indirect effects 1, 2, and 3) was significant: its confidence interval was entirely above zero (95% bootstrap CI = [0.072, 0.138]). Finally, as in our previous analyses of simple mediation, the direct effect (path C'; *β* = 0.094) was smaller than the total effect (path C; *β* = 0.198) but still remained statistically significant (*p* = 0.004).

From our study using adaptive tasks in a large sample of preschool children aged from 3 to 6 (as early as possible in their educational curriculum), we conclude that emotion knowledge, social behaviour, locomotor activity, and academic-mathematic performance are interrelated. Mediation analyses revealed that higher academic-mathematic performances are associated with higher scores in both emotion knowledge and social behaviour and, in turn, children with a greater comprehension of emotions tend to have higher locomotor and academic-mathematic skills (Fig. 2b). Additionally, the sequential mediation analysis supports and complements these findings by showing that differences in emotion knowledge, locomotor activity and social behaviour explain, at least partially, academic-mathematic performance (Fig. 2c).

The present data extend previous work in this area in a number of important ways. First, very few studies have focused specifically on the effect of emotion knowledge on mathematic-numerical abilities of preschool children. Second, no study has directly examined the importance of locomotor activity in the prediction of emotion knowledge and academic-mathematic performance in preschool years. Third, no study has so far been performed on a large cohort of children in preschool. Furthermore, and in light of the recent debate about reproducibility, the side goal of our study was to assess whether the published results were found to be true in another educational context^30^.

Regarding the emotion knowledge assessed in the present study, it was established that this ability contributes to the regulation of emotions and behaviour^31^, which in turn influences children's academic success^32^ as well as the development of appropriate and adaptive social behaviour during the preschool years^33^. A meta-analysis review highlights the relations between emotion knowledge and social competence^1^. More particularly, some researchers showed that social competence mediated the association between early social understanding (including belief understanding and emotion understanding) and later school achievement^12^. In line with these studies, the present data support the mediation role of social behaviour between emotion knowledge and academic-mathematic performance.

Even though the role of emotion and affective processes on learning is not new (with for example the concepts of “punishment” and “reward”), this is a relatively new field of investigation^34,35^. Studies have shown that emotions have an impact on students’ well-being at school, on their learning abilities, and on the achievement of academic goals^5,10,36^. Positive emotions can represent a powerful catalyst for school well-being and academic success, but negative ones can be a vector of failure and loss of trust when they are inappropriate, deregulated or ignored. Pekrun (1992) criticizes the fact that research in the educational domain is mainly focused on anxiety, neglecting other emotions that are supposed to exert negative or facilitating influences on achievement and to impact the cognitive strategies used in the learning process and motivation^37^.

With regard to locomotor activity, the results support that it significantly predicts academic-mathematic performance. This result is in line with the meta-analysis that determined a positive relation between physical activity and cognitive performance in school-age children, particularly in early elementary age children and in middle school^38^. One explanation could be that physical activity in children may impact the development of brain structures modifying white matter integrity and activation of regions key to cognitive processes (see Ref.^39^ for a review). This would explain the effect of this variable on children's academic-mathematic performance. Studies conducted with adult subjects also show that physical activity enhances cognition and more specifically the functions of executive control^40,41^. In a one-year follow-up research, executive functions and motor coordination skills of children (between 4 and 6 years old) with and without motor impairments were examined. The results highlight that children with persisting motor coordination impairments showed weaker inhibition/interference control and weaker performance in visuospatial static and dynamic working memory^42^. From a neuroscientific perspective, results showed that executive functions in adults are related to a set of distributed frontal cortico-cerebellar regions that include some areas also implicated in early development of motor skills^43^. These findings highlight that children with delayed motor development may show relatively poor executive functions as adults, particularly because executive functions are essential in the development of coordinated movements.

Our results also reveal that locomotor skills have an effect on children's social behaviour and on their ability to understand emotions. In addition, emotion comprehension and social behaviour mediate the link between locomotor activity and academic-mathematic performance, respectively. A possible explanation for this result may lie in the fact that these locomotor skills are essential to enable children to engage in social play, especially at school and particularly in those age groups where locomotor activity is important^14^. Indeed, locomotor activity would be a privileged way of interacting with others and experiencing emotions, whether in more dynamic play such as chasing, running, jumping, climbing, and rough-and-tumble or in forms of play such as make-believe where children are required to adapt and manage their movements according to the roles chosen and the evolution of the play scenario. Results demonstrated that kindergartener children with low motor abilities are less likely to engage in social play and exhibit higher frequency of social reticence during free play^23^. These more reticent children, in turn, would have fewer relational opportunities to develop their understanding of emotions and, consequently, social behaviour that would impede them to maintain positive relationships. Thus, being socially competent represents an essential support to benefit from assistance from others when the child is learning more academic skills such as mathematics. As Zins, Bloodworth, Weissberg and Walhberg (2007) point out: “Intrinsically, schools are social places and learning is a social process.” (p. 191)^44^. In conclusion, our findings are consistent with the political and scientific consensus on the importance of social-emotional competences in the academic world at the beginning of school^45–47^ and suggest adding locomotor activity to these foundational abilities.

Finally, we aim to distinguish three mechanisms underlying these different outcomes. The first mechanism of the relation between emotion knowledge and academic-mathematical achievement mediated by social behaviour refers to what we have just mentioned above, namely the fact that to learn and develop, children (especially young children) need adults and peers. This can be conceptualized as the zone of proximal development (ZPD). In a cultural-historical view, the ZPD refers to what children can achieve in collaboration with others in relation to children's current and future developmental period^48^. The link with emotion understanding is then the following: children with better knowledge of their emotions would be more able to regulate them^32^. Better management of these emotions would facilitate social interactions, allowing children to form better relationships with teachers, peers, and family. This may also indirectly influence academic achievement through providing pupils with a “social support network” that protects them in times of stress and supports them when they are confronted with a new situation or learning challenge requiring expert (peer, teacher) help^10^.

The second mechanism explaining the link between emotion knowledge and academic-mathematical achievement could be related to the overlap between emotion knowledge and intellectual competencies^10^. As the authors note, having knowledge about emotions could be part of a sub-dimension of verbal knowledge/ability. Learning new emotion words may in fact result in learning academic competencies (such as spelling, vocabulary). Thus, children who are good at emotion understanding may also be good at other areas at school (such as language or number acquisition).

The third mechanism explaining the relation between locomotor activity and academic-mathematical achievement could be related, as mentioned above, to the development of executive functions and more particularly of inhibitory control. Preschool years are a sensible period for this development^49–54^. Indeed, it is during this period that core components of executive functions develop, forming a crucial foundation that will set the stage for the development of higher cognitive processes well into adulthood^54^. The development of inhibitory control, which includes the resistance to distracter interference (interference control) and inhibition of action (inhibiting a prepotent response)^55^ will allow children to exercise more voluntary control over their thoughts and behaviour. As a result, inhibitory control will play a central role in children's social and academic adaptation as well as in their emerging academic skills^55,56^, particularly mathematical skills^57^. Interestingly, in this meta-analysis, inhibitory control was more strongly associated with early math skills than with early literacy skills. In this vein, in 7- to 11-year-old children, physical activity shows benefits particularly on executive function and mathematic achievement with no benefit to reading. In this same study, children who benefited from the exercise program showed increased prefrontal cortex activity and reduced posterior parietal cortex activity^58^. In the neuroscientific perspective, some studies showed an overlap in the neural substrates supporting executive function, numerical ability, and quantitative reasoning^56^. We then assume that locomotor activities (frequent during this period) constitute a privileged way for young children to progressively learn to control their own motor behaviour and to control their emotions when they are engaged in locomotor play (such as rough and tumble or physical play). Various studies have thus shown that physical activity is positively related to the cognitive development of young children^38^.

A study examined whether higher levels of active play, defined by authors as play that incorporates movement at a moderate to vigorous intensity, predicted better self-regulation in 51 prekindergarten children. Path analyses demonstrated that higher active play was associated with better self-regulation, which in turn was associated with higher emergent literacy and math scores^59^. Some sports such as Tae Kwon Do could improve self-regulatory abilities from kindergarten to grade 5^60^. Encouraging locomotor activities and making place for movement, particularly at school, would improve inhibitory control and thus promote the ability to inhibit prepotent response tendencies and distracting information when the child resolves, for instance, a math problem and foster better emotion management, which in turn, as indicated in the first explanatory mechanism, would enable children to acquire new academic knowledge.

From the application point of view, these results have important implications at the level of teaching and education. Indeed, they support the idea that it is essential to provide a pedagogical structure adapted to the way children learn at the beginning of school through two means. The first is giving a privileged status to “play” (such as constructing play, make believe play, and locomotor play), that fosters relational opportunities with peers, allows for the reinvestment, experimentation and consolidation of knowledge such as numerical abilities, social-emotional competences^61^, and gives an essential place for movement. The second means is providing teaching time centred on these different specific abilities and forms of knowledge inside and outside these moments of play.

The main limitation of this study is its cross-sectional design. Without longitudinal data nor post-training data, we are limited to drawing causal inferences about the links among the development of emotion knowledge, social behaviour, locomotor activity, and numerical abilities. Although mediation models provide statistical support for the claim that emotion knowledge, locomotor activity, and social behaviour “explain” academic-mathematic performance, this technique is still correlational in nature. Our results have demonstrated that emotion knowledge, social behaviour, locomotor activity, and academic-mathematic performance are significantly associated but not that these abilities cause higher achievement.

Finally, one direction for further research is to evaluate the effects of different training interventions based on these respective abilities on the development of academic-mathematic performance in preschool children. This design would isolate which abilities are most relevant for the improvement of mathematic performance and would also provide stronger evidence for the causal directions from these abilities to academic performance.

## Methods

The study (including experimental protocol and data collection) was conducted in accordance with ethical principles for research involving human subjects (World Medical Association Declaration of Helsinki) and was approved by the DSDEN-73 committee and its Academic Director (DASEN) of French National Education (MEN). The study carried out in France was also conducted in accordance with the Swiss recommendations of the ethic committee of the Faculty of Psychology and Educational Sciences (FPSE) from the University of Geneva. A written informed consent of each child’s parent was obtained from all participants.

### Participants

In total, 706 children (age range: 35 to 71 months, *M* = 54.35, *SD* = 10.41; 354 girls) were retained for the study. They were recruited in 33 classes from 27 rural (< 1000 inhabitants; *n* = 271), village (1000–5000 inhabitants; *n* = 260), and urban (5001 to + 20′000 inhabitants; *n* = 175) public preschools located over a very large area of Savoie Department (France, 73). These were single-, double- and triple-grade classes, taught by experienced teachers, with between 8 and 29 pupils per class (see Supplementary Table S1 for details). Children were all French-speaking and came from families with low to high SES. Data from children reported to have special educational needs because of learning difficulties or any other disability (e.g., deafness, Down's syndrome, etc.) (*n* = 10), with scores on the different tasks less or greater than 3 standard deviations from the participants’ mean scores (*n* = 7), as well as data that encountered procedural errors in their collection (*n* = 11) were excluded from the study. Children were tested in schools by their teachers who had been previously trained and continuously monitored via a digital platform. The assessments of academic-mathematic performance and emotion knowledge were conducted under normal classroom conditions (with a preliminary pilot experiment to test the emotion task’s protocol we designed in order to verify its feasibility and validity). An assistant was specially hired to teach and manage the class while the teacher tested each pupil individually. Social behaviour and locomotor activity were assessed collectively during physical education classes. All evaluations took place in the first months of the school year (between October and November 2019). Such collaboration with the teachers provided dual benefits. First, we were able to provide them with an experimental protocol along with the specific material, instructions and scoring guidelines required for its implementation to test the children's emotion knowledge. Second, we could rely on the official teaching aids by integrating part of their content into the present study in order to assess other competencies while complying with the preschool objectives (thus we avoided disrupting the teaching curriculum for the classes involved in the study).

### Study overview

This study comprised four main measures: (1) emotion knowledge, evaluated with an emotion comprehension task that we had created (see Supplementary Material) and pre-tested before providing it to teachers who had been previously trained to implement it in their classrooms under standardized conditions; (2) academic-mathematic performance scores, computed from three numerical tasks that we had selected in accordance with the teachers among the various mathematical exercises proposed by the official teaching aids; (3) social behaviour, referred to children's reactions and attitudes when playing two different team games (one with ball and the other without) observed by teachers and reported via an adapted version of the evaluation grid provided by the official preschool teaching materials that included items specifically applicable to the selected collective games; and (4) locomotor activity, also measured through teachers’ observation of the children's performances during the implementation of an agility trail, rated using the scoring grid that we have adapted based on the content of the official teaching aids (cf. Supplementary Material).

### Academic-mathematic performance

Academic-mathematic performance was assessed by three numerical tasks (underlying distinct early numerical abilities) taken from the official teaching aids.

The first task concerned the construction of numbers to express quantities. Its aim was to assess whether the children had understood that the cardinal of a collection does not change when the spatial arrangement or the nature of its elements is modified. For this purpose, the teacher placed a photograph in front of each pupil showing four collections of objects, two of which had the same number of items (e.g., one pair of scissors, two tokens, two pens and three tubes of glue). Each child was then asked to point (1) to the collection that had the *most* items, (2) to the one that had the *least*, and (3) to the two collections that had the *same* number of items. If a child managed to answer easily, the teacher placed another photograph in front of him/her with collections of more elements (e.g., three tubes of glue, four brushes, four pairs of scissors and five tokens). As with the previous picture, the child was invited again to point to the collection with the most objects, to the one with the fewest, and to the two with the same number.

The second task assessed children's ability to use numbers to designate the rank of an item in an ordered sequence. To do this, the teacher placed ten tokens (e.g., five red and five yellow) in a row in front of each pupil (lining them up in the reading direction), then asked him/her to point (1) to the first token, (2) to the last token, (3) to the 3rd token in the row, (4) to the 5th token in the row, (5) to the 2nd yellow token, and (6) to the last red token. Nevertheless, the teacher did not necessarily perform the entire exercise with each pupil: when a child was no longer able to answer one of these questions correctly, the task was completed (i.e., the questions of higher difficulty than the child's levels were not assessed).

The third task examined the children's ability to identify the organizing principle of an algorithm by (a) continuing its application, and by (b) filling in gaps in organized sequences. Therefore, this last task was carried out in two main steps: first of all, the teacher began by successively producing several algorithmic sequences (of increasing difficulty) composed of several copies of two (A-B) and then three (A-B-C) objects (30 in total: *n*_A_ = 14, *n*_B_ = 10, *n*_C_ = 6) of different shapes and colours, aligned in front of the pupil in the reading direction. Each child was invited then to attempt to pursue the following sequences: ABAB[…], ABCABC[…], AABAAB[…], AABCAABC[…], AAABBCAAABBC[…]. The second step of this task consisted in presenting the pupil with up to six (as long as he or she did not fail) new algorithmic sequences which, this time, were incomplete (,,,,,). The teacher deliberately left these gaps in order to ask the child to fill them in with the correct shape and colour objects. In both the first and second of these exercises, the task was completed when the child was no longer able to pursue one of the assessed algorithmic sequences (tested in order of increasing difficulty in the first step), and when he or she failed to fill in the gaps in one of the new sequences that had also been successively presented to him/her in the second step.

Given that the performance of each of these three tasks measuring children's early numerical abilities depended on the level of each one (items of higher difficulty than the failed level were not performed), the maximum score that could be obtained for each of them varied. For this reason, we decided to express each child's overall academic-mathematic performance as a percentage of success computing from the results rather than calculating a total score.

### Emotion knowledge

Emotion knowledge was assessed by an emotion comprehension task (adapted from previous work)^27,28^ comprising two main sub-tasks: (1) the recognition of the primary emotions of anger, fear, joy and sadness as well as a neutral facial expression, and (2) the comprehension of the external causes underlying these emotions in others, itself subdivided into two different tasks: (a) to point to which of these five emotions a character feels in several given situations, and (b) to name each of them correctly. The detailed content of this task, the test sheet, and its scoring key are available in the Supplementary Information file (cf. Material) to this manuscript.

The first main sub-task consisted in recognizing an emotion on the basis of facial expression. Each child was invited to match an emotion label previously heard (word said by the teacher) to the corresponding emotional facial expression from a choice of five answers. Five pictures illustrating four facial expressions of primary emotions (angry, fear, happy, sad) and a neutral expression were randomly presented by the teacher. Subsequently, the participants were invited to give the teacher the picture of the child “who feels happy” for example. The pupil was invited to choose the right answer. The maximum score for this task is 5 points (one point per correct answer).

The second main sub-task consisted in understanding the causes of these same emotions by (a) pointing to, and (b) naming the one that corresponds to the emotion felt by a character in five given situations based on external contextual elements. The teacher presented five cartoon scenarios illustrated by a picture of a protagonist facing a particular situation (e.g., "A boy just got a birthday present"). While showing a given scenario, the teacher read the story about the character depicted. The face of the character in the picture was left blank. Each of these situations could induce four emotional responses (angry, sad, happy, fear) plus one neutral. For each board, the child was asked to answer how the protagonist felt in that situation, first by pointing (non-verbal responses) to one of the five illustrations representing the facial expressions of the character’s emotional responses and the neutral response mentioned above, and subsequently, by labelling the emotion felt by the character. The maximum score for each of these two sub-tasks was 5 points (one point per correctly identified item and one point per correctly labelled emotion).

By summing up the points obtained in the three sub-tasks (*recognizing*, *pointing to,* and *labelling* emotions), we then calculated each child’s emotion knowledge total score (range: 0 to 15 points).

### Locomotor activity

Children’s locomotor activity was evaluated through the completion of an agility trail made up of various installations arranged on the ground and at height. In order to measure each child's performance on this trail, a scoring grid was developed in collaboration with the teachers on the basis of the official teaching aids to ensure that it would be adapted to both the youngest and oldest pupils involved in the study. Altogether, this grid included items assessing the following nine locomotor skills: balancing, climbing, crawling, crossing an obstacle in height and in width, hanging, swinging, sliding, and throwing. The detailed scoring grid is available in the Supplementary Information file (cf. Material) to this manuscript. However, due to material constraints, not all teachers were able to test their students in each of the nine competencies (e.g., in some rural schools, physical education classes were held exclusively outdoors: since these establishments did not have gymnasiums, teachers from such locations often did not have adequate equipment to assess all areas of the grid). Therefore, the minimum number of areas to be tested was fixed at five. Despite the use of this scoring grid, individual total scores could not be computed because the number of locomotor skills assessed varied across schools. For this reason, we calculated the ratio (expressed as a percentage of success) between the sum of points obtained by each child and the maximum possible points which depended on the number of areas tested but also on their rating: balancing and crawling were scored on two points while the seven other skills were scored on three points.

In addition to the different locomotor skills, the grid also included five questions about the child's overall attitude towards the whole activity, to which all teachers responded (“yes” = 1 point, “no” = 0 point). Through these items, they assessed whether the pupil… (1) agreed to take the agility trail proposed; (2) attempted to complete each obstacle (with or without assistance); (3) evolved his actions between the obstacles to better overcome them; (4) dared to venture into acrobatic or unbalanced situations despite his fear; (5) was satisfied with his performance on the trail and proud of his personal accomplishment. Each child was allowed to complete the entire agility trail twice, only the best attempt was evaluated.

### Social behaviour

Children's social behaviour was assessed through the social actions displayed when playing collective games during a regular physical education course. A total of six team games (three ball games and three ball-less games) were selected among the physical activities list of the official teaching aids to measure social behaviour in the present study (illustrations of these set-ups are available online here: http://www.petitsateliers.fr/sport/ateliers/).

Teachers were asked to choose both a ball game (between “Protect the castle”, “Hot potato”, and “Dodgeball”) and a non-ball game (between “Colour run”, “The moving circles”, and “Hens foxes snakes”) according to their class level in order to assess the social behaviour of their pupils when playing each of these games. To do so, teachers used an adapted version of the evaluation grid provided by the official teaching aids covering the following six dimensions through specific sub-items for each game: (1) the child engaged in the activity proposed by the teacher (“yes” = 1 point, “no” = 0 point); (2) the child understood and accepted the rules of the game (from two to five rules, depending on the game, each one rated either 1 or 0 point); (3) the child implemented these rules and acted in accordance with them in order to achieve a collective objective (1 point per rule followed); (4) the child has recognized his/her belonging to a given team (1 point), and has played several complementary roles to promote that team and lead it to victory (two to three roles, depending on the game, 1 point each); (5) the child found his/her location in a space designed to oppose the plan of an adversary or an opposing team (1 point); and (6) the child developed individual or collective strategies to find the most effective ways of doing things (1 point). The maximum number of points that could be obtained with this scoring grid varied according to the complexity of the selected games, thus hindering the comparison of pupils' total scores. Consequently, we again opted to calculate each child's percentage of success.

Finally, the composition of the teams was not the same for the two types of games in order to minimize the influence of teammates on individual social behaviours.

### Data analysis

Given that teachers conducted the assessments during their classes (under regular classroom conditions) and that some tasks were based on their observations (especially the social behaviour and locomotor activity tasks and the emotion knowledge's labelling sub-task), the data they collected were blind-coded by two different experimenters before being statistically analysed. Statistical analyses were computed using both TIBCO Statistica 13.2 and IMB SPSS Statistics 26.0 computer software. A *p* value ≤ 0.05 was considered significant.

#### Relations between the different measures

We performed correlation analyses (partial Pearson’s *r*) among emotion knowledge total score, social behaviour, locomotor activity, academic-mathematic performance scores (in percentages) and participants’ age in months (integrated as a continuous independent variable in the correlation matrix) while controlling for the gender, living location, and assessing teacher independent variables.

#### Developmental shifts in all variables

We used simple linear regression analyses to test for linear differences in academic-mathematic performance, emotion knowledge, social behaviour and locomotor activity across age.

#### Predictors of academic-mathematic performance

We investigate whether differences in academic-mathematic performance across age could be better predicted by our hypothesized predictors (i.e., emotion knowledge, social behaviour, and locomotor activity) but also by the independent variables considered in the previous correlation analyses (i.e., gender, living location, and assessing teacher). We performed a stepwise regression procedure by entering these seven predictors into the analysis (in one block) in order to determine the best predictive model accounting for differences in academic-mathematic performance. Before proceeding with this analysis, we verified that our data met the assumptions associated with multiple linear regression (linear relation multivariate normality, little or no multicollinearity, no auto-correlation, homoscedasticity of error variance).

#### Mediation analyses

The mediation analyses were conducted following the same statistical logic adopted by various authors in recent previous studies^12,13,26,62^. We used the PROCESS macro (Version 3.5.3) for SPSS, Model 4^63^ to test both of our simple mediation models, which assumed that (a) social behaviour mediated the effect of emotion knowledge on mathematics, and (b) emotion knowledge mediated the effect of locomotor activity on academic-mathematic performance scores. Percentile bootstrap confidence intervals of 95% for the mediation model were obtained using 5′000 bootstrap samples. These analyses were run with age in months (continuous variable) entered as covariate in order to control for the effect of the age while evaluating the potential mediation effects. We then became interested in testing whether these two simple mediation models could merge into a multiple mediation model. For this purpose, we used PROCESS macro for SPSS, Model 6^63^ to perform a sequential mediation with two mediators (M1, emotion knowledge; M2, social behaviour) still controlling for the age in months (entered as covariate in the model) in order to better understand the relation between locomotor activity and academic-mathematic performance. In the end, it turned out that these analyses only revealed “partial mediation effects” (as argued by some experts) between our variables through the different models tested. Although the classical approach (i.e., the “causal steps strategy”^64^) stipulates that the effect of X on Y (c') should no longer be significant when a mediating variable is entered into the model, some authors^65–67^ have demonstrated that this significant decrease (of the direct effect c' compared to the total effect c) is not necessary for mediation to occur. Additionally, a direct effect being exactly zero does not preclude there from being other mechanisms by which the independent variable influences the outcome variable^68,69^. Significant mediation at an α level of 0.05 was defined as 95% CI_s_ of the indirect effects that did not include 0. All tests were two-sided. Prior to conducting these mediation analyses, the *independence of observation assumption* was investigated by calculating an ICC (of 9.69%) using the mixed model procedure in SPSS. This analysis was conducted with a fully unconditional model in which the assessing teacher was entered as a random effect variable.

## Supplementary Information

Supplementary Information 1.

Supplementary Information 2.

Supplementary Information 3.
