# Supplementary material for: Emotion knowledge, social behaviour and locomotor activity predict the mathematic performance in 706 preschool children
Source: Sci Rep. 2021 Jul 13;11:14399. doi: 10.1038/s41598-021-93706-7 (PMC8277886; doi:10.1038/s41598-021-93706-7)
Supplement: Supplementary file 1 — Supplementary Information 1. [file 41598_2021_93706_MOESM1_ESM.docx]

**Electronic supplementary material**

[***Supplementary Information***](https://static-content.springer.com/esm/art%3A10.1038%2Fs41562-017-0238-7/MediaObjects/41562_2017_238_MOESM1_ESM.pdf) ***file.*** Supplementary Table S1, Supplementary Figure S1, Supplementary Materials

***Supplementary Data.*** Data for this report are available through this file for full scientific transparency in the Supplementary Data file.
