# Supplementary material for: Emotion knowledge, social behaviour and locomotor activity predict the mathematic performance in 706 preschool children
Source: Sci Rep. 2021 Jul 13;11:14399. doi: 10.1038/s41598-021-93706-7 (PMC8277886; doi:10.1038/s41598-021-93706-7)
Supplement: Supplementary file 2 — Supplementary Information 2. [file 41598_2021_93706_MOESM2_ESM.docx]

**Emotion knowledge, social behaviour and locomotor activity predict the mathematic performance in 706 preschool children**

**Thalia Cavadini^1^, Sylvie Richard^1,2^, Nathalie Dalla-Libera^3^& Edouard Gentaz*^1,4,5^**

^1^University of Geneva, Department of Psychology, Switzerland

^2^Valais University of Teacher Education, Switzerland

^3^Minister of National Education, Savoie Department, France

^4^Swiss Center for Affective Sciences, University of Geneva Switzerland

^5^CNRS, Grenoble, France

* Corresponding author: Prof. Edouard Gentaz, Email: [Edouard.Gentaz@unige.ch](mailto:Edouard.Gentaz@unige.ch)

**Supplementary Information**

[Supplementary Table 2](#_Toc65519617)

[Supplementary Figure 4](#_Toc65519618)

[Supplementary Material 5](#_Toc65519619)

[1. Emotion Comprehension Task Content 5](#_Toc65519620)

[Sub-task 1: Recognition of the primary emotions 5](#_Toc65519621)

[Sub-task 2: Comprehension of the primary emotions’ external causes 8](#_Toc65519622)

[2. Emotion Comprehension Task Test Sheet 13](#_Toc65519623)

[Sub-task 1: Recognition of the primary emotions 13](#_Toc65519624)

[Sub-task 2: Comprehension of the primary emotions’ external causes 13](#_Toc65519625)

[3. Emotion Comprehension Task Scoring Key 14](#_Toc65519626)

[4. Locomotor activity: Scoring grid 15](#_Toc65519627)

# Supplementary Table

| *Table S1*. Preschool locations and distribution of participants by grades, classes and schools | | | | | | | |
| --- | --- | --- | --- | --- | --- | --- | --- |
|  | **School location^1^** | **Class number** | ***n*** | | | |  |
|  |  |  | 1^st^ grade | 2^nd^ grade | 3^rd^ grade | per class | **Total** |
| School 1 | Village | Class 1 | 5 | 5 | 7 | 17 | **17** |
|  |  |  |  |  |  |  |  |
| School 2 | Rural | Class 2 | 14 | 13 | 0 | 27 | **53** |
|  |  | Class 3 | 0 | 10 | 16 | 26 |  |
|  |  |  |  |  |  |  |  |
| School 3 | Village | Class 4 | 0 | 6 | 10 | 16 | **16** |
| School 4 | Village | Class 5 | 0 | 0 | 26 | 26 | **26** |
| School 5 | Rural | Class 6 | 6 | 10 | 13 | 29 | **29** |
| School 6 | Village | Class 7 | 15 | 9 | 0 | 24 | **24** |
| School 7 | Village | Class 8 | 0 | 12 | 15 | 27 | **27** |
| School 8 | Rural | Class 9 | 10 | 10 | 6 | 26 | **26** |
| School 9 | Rural | Class 10 | 0 | 0 | 19 | 19 | **19** |
| School 10 | Rural | Class 11 | 4 | 4 | 4 | 12 | **12** |
| School 11 | Rural | Class 12 | 4 | 1 | 3 | 8 | **8** |
|  |  |  |  |  |  |  |  |
| School 12 | Urban | Class 13 | 6 | 8 | 1 | 15 | **31** |
|  |  | Class 14 | 5 | 5 | 6 | 16 |  |
|  |  |  |  |  |  |  |  |
| School 13 | Rural | Class 15 | 6 | 5 | 6 | 17 | **17** |
| School 14 | Village | Class 16 | 23 | 0 | 0 | 23 | **23** |
| School 15 | Rural | Class 17 | 0 | 6 | 4 | 10 | **10** |
| School 16 | Urban | Class 18 | 0 | 8 | 20 | 28 | **28** |
| School 17 | Rural | Class 19 | 4 | 9 | 0 | 13 | **13** |
| School 18 | Village | Class 20 | 0 | 0 | 25 | 25 | **25** |
| School 19 | Urban | Class 21 | 7 | 7 | 10 | 24 | **24** |
|  |  |  |  |  |  |  |  |
| School 20 | Rural | Class 22 | 23 | 0 | 4 | 27 | **54** |
|  |  | Class 23 | 0 | 17 | 10 | 27 |  |
|  |  |  |  |  |  |  |  |
| School 21 | Village | Class 24 | 17 | 11 | 0 | 28 | **56** |
|  |  | Class 25 | 18 | 10 | 0 | 28 |  |
|  |  |  |  |  |  |  |  |
| School 22 | Village | Class 26 | 5 | 9 | 9 | 23 | **23** |
|  |  |  |  |  |  |  |  |
| School 23 | Urban | Class 27 | 0 | 9 | 12 | 21 | **45** |
|  |  | Class 28 | 24 | 0 | 0 | 24 |  |
|  |  |  |  |  |  |  |  |
| School 24 | Urban | Class 29 | 0 | 9 | 15 | 24 | **47** |
|  |  | Class 30 | 0 | 0 | 23 | 23 |  |
|  |  |  |  |  |  |  |  |
| School 25 | Rural | Class 31 | 8 | 11 | 0 | 19 | **19** |
| School 26 | Village | Class 32 | 0 | 4 | 19 | 23 | **23** |
| School 27 | Rural | Class 33 | 2 | 3 | 6 | 11 | **11** |
|  |  | **Total** | **206** | **211** | **289** | **706** |  |
| ***Note.***  *^1.^* Rural: areas of less than 1000 inhabitants, Village: areas of 1000 to 5000 inhabitants, Urban: areas of 5001 to 20'000 and over inhabitants | | | | | | | |

# Supplementary Figure


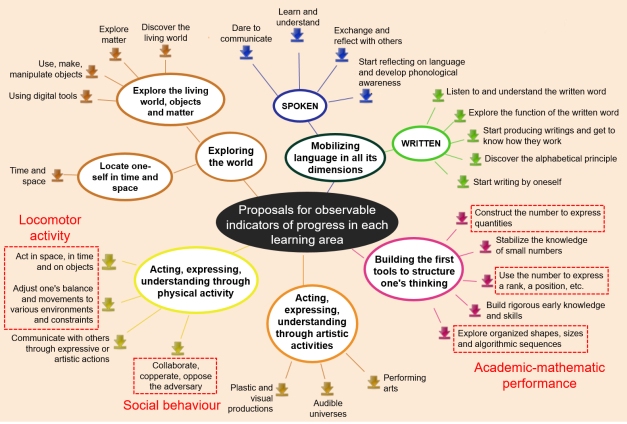


Figure S1. **A schematic representation of the five official learning domains in French preschools.** The competencies of interest for the present study are indicated in red.

# Supplementary Material

## Emotion Comprehension Task Content

### Sub-task 1: Recognition of the primary emotions

**Instructions** (the experimenter sits next to the child): *"Today I would like to understand how children of your age can feel in life, at home or at school. So we're going to do an activity together.*

- *I'm going to start by asking you some questions.*
- *Then I'm going to show you some pictures. You will have to look at the pictures carefully.*
- *Then you will have to choose one.*
- *On this sheet of paper I will write down what you say. There is no right or wrong answer! Just answer as you think.”*

*1 Look at these pictures. Point to the picture of the child who feels* ***happy****.*


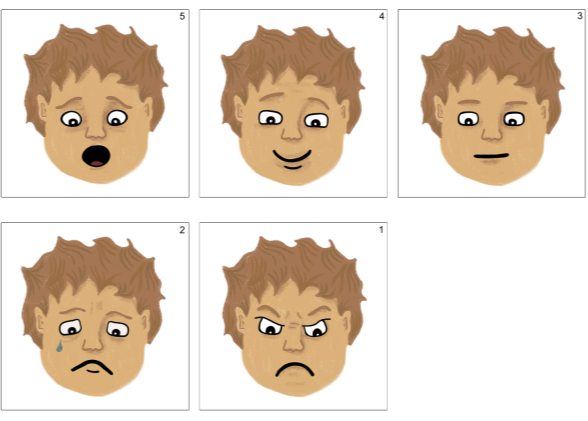


1. *Look at these pictures. Point to the picture of the child who feels* ***sad****.*


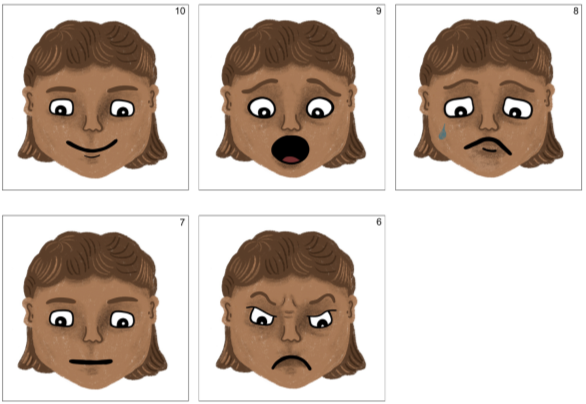


1. *Look at these pictures. Point to the picture of the child who feels* ***angry****.*


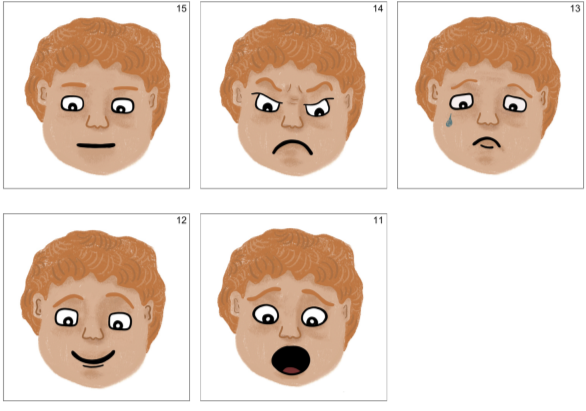


1. *Look at these pictures. Point to the picture of the child who feels* ***alright****.*
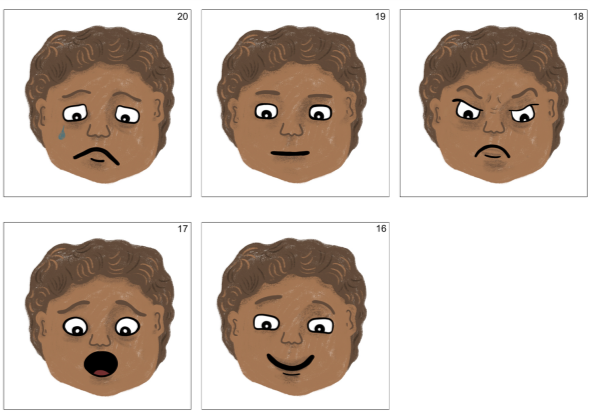

2. *Look at these pictures. Point to the picture of the child who feels* ***scared****.*


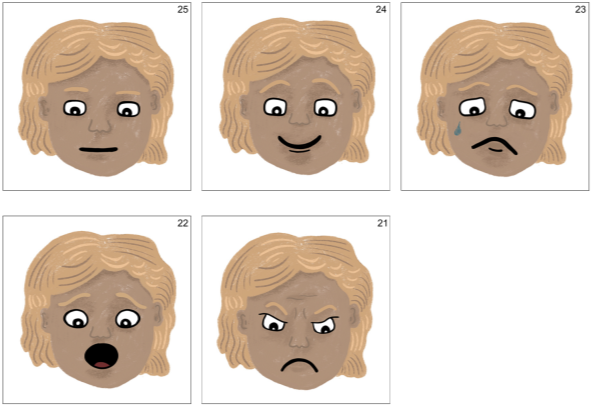


### Sub-task 2: Comprehension of the primary emotions’ external causes

**Instructions** (the experimenter is still sitting next to the child): *"Now you will see some other pictures. Each time, I'm going to tell you a short story. Listen to the story carefully until the end before you give your answer.”*

1. ***That boy just got a birthday present.***
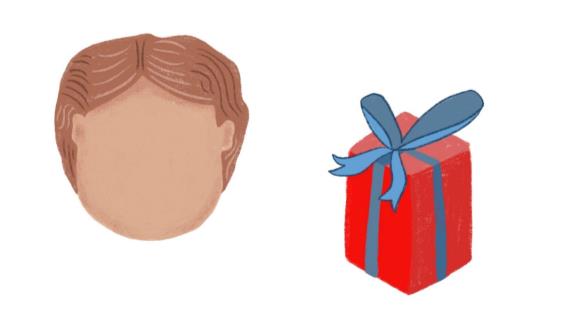

2. *How is this boy feeling? Show me the picture that matches his face.*


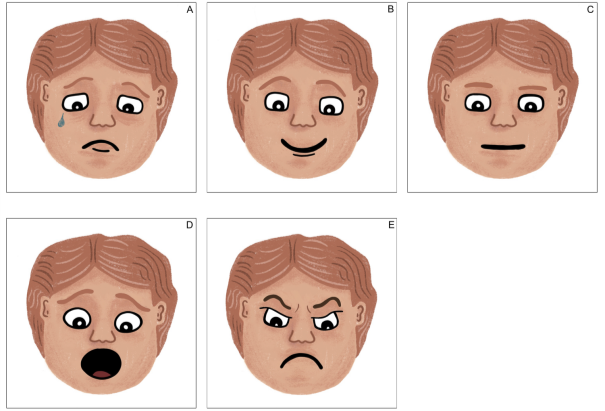


1. *How does he feel at that moment?*
2. ***This girl is looking at her fish, which is very sick.***


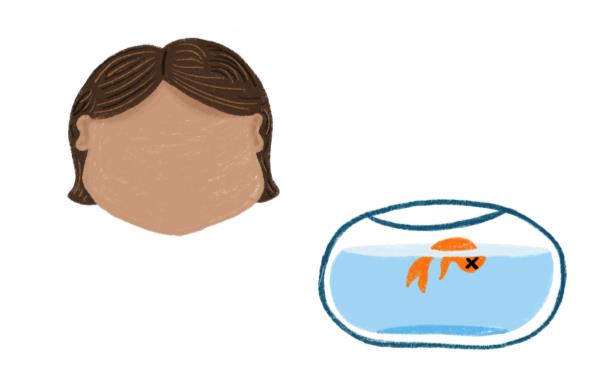


1. *How is this girl feeling? Show me the picture that matches her face.*


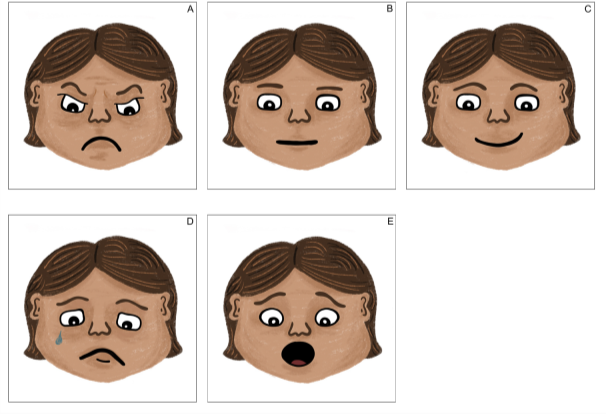


1. *How does she feel at that moment?*
2. ***This girl is trying to make a nice drawing but her little sister deliberately spills a glass of apple juice on it.***


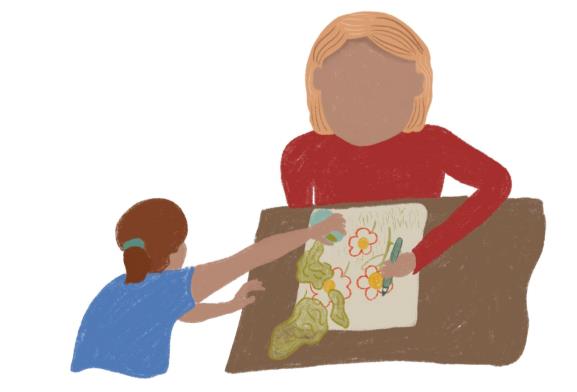


1. *How is this girl feeling? Show me the picture that matches her face.*


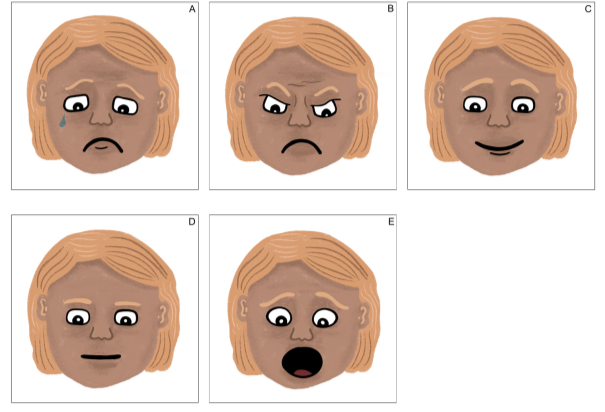


1. *How does she feel at that moment?*
2. ***This boy just woke up on a regular morning.***


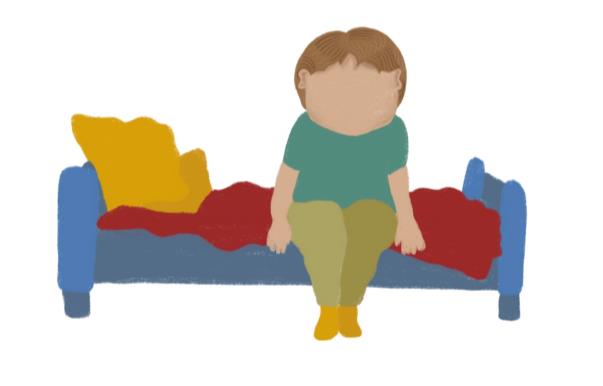


1. *How is this boy feeling? Show me the picture that matches his face.*


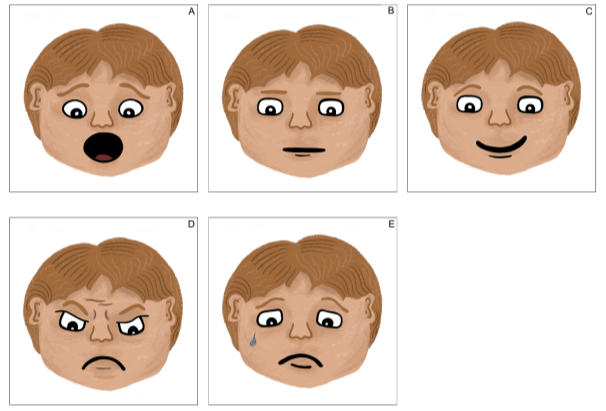


1. *How does he feel at that moment?*
2. ***This boy is being chased by a monster.***


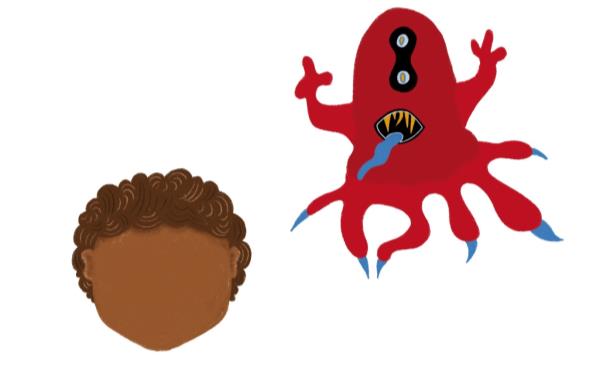


1. *How is this boy feeling? Show me the picture that matches his face.*


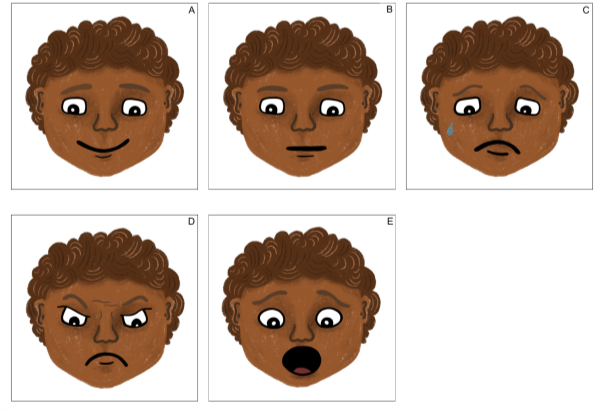


1. *How does he feel at that moment?*

## Emotion Comprehension Task Test Sheet

First name……………………………………… Class/school grade……………… Date…………………

### Sub-task 1: Recognition of the primary emotions

**Instructions** (the experimenter sits next to the child): *"Today I would like to understand how children of your age can feel in life, at home or at school. So we're going to do an activity together.*

- *I'm going to start by asking you some questions.*
- *Then I'm going to show you some pictures. You will have to look at the pictures carefully.*
- *Then you will have to choose one.*
- *On this sheet of paper I will write down what you say. There is no right or wrong answer! Just answer as you think.”*

| Item | Question ? | Cards  (randomly arranged) | | | | | Answers given (to note) |
| --- | --- | --- | --- | --- | --- | --- | --- |
| 1 | *Look at these pictures. Point to the picture of the child who feels* ***happy****.* | 1 | 2 | 3 | **4** | 5 |  |
| 2 | *Look at these pictures. Point to the picture of the child who feels* ***sad****.* | 6 | 7 | **8** | 9 | 10 |  |
| 3 | *Look at these pictures. Point to the picture of the child who feels* ***angry****.* | 11 | 12 | 13 | **14** | 15 |  |
| 4 | *Look at these pictures. Point to the picture of the child who feels* ***alright****.* | 16 | 17 | 18 | **19** | 20 |  |
| 5 | *Look at these pictures. Point to the picture of the child who feels* ***scared****.* | 21 | **22** | 23 | 24 | 25 |  |
| Sub-task 2: Comprehension of the primary emotions’ external causes **Instructions** (the experimenter is still sitting next to the child): *"Now you will see some other pictures. Each time, I'm going to tell you a short story. Listen to the story carefully until the end before you give your answer.”* | | | | | | | |
| 6 | ***This boy just got a birthday present.*** | Large card 1 | | | | | a) Pointing: |
|  | a) *How is this boy feeling? Show me the picture that matches his face.* | A | **B** | C | D | E | ………………….. |
|  | b) *How does he feel at that moment?* |  |  |  |  |  | b) Labelling:  ………………….. |
| 7 | ***This girl is looking at her fish, which is very sick.*** | Large card 2 | | | | | a) Pointing: |
|  | a) *How is this girl feeling? Show me the picture that matches her face.* | A | B | C | **D** | E | ………………….. |
|  | b) *How does she feel at that moment?* |  |  |  |  |  | b) Labelling:  ………………….. |
| 8 | ***This girl is trying to make a nice drawing but her little sister deliberately spills a glass of apple juice on it.*** | Large card 3 | | | | | a) Pointing: |
|  | a) *How is this girl feeling? Show me the picture that matches her face.* | A | **B** | C | D | E | ………………..…b) Labelling: |
|  | b) *How does she feel at that moment?* |  |  |  |  |  | ………………….. |
| 9 | ***This boy just woke up on a regular morning.*** | Large card 4 | | | | | a) Pointing: |
|  | a) *How is this boy feeling? Show me the picture that matches his face* | A | **B** | C | D | E | ………………….. |
|  | b) *How does he feel at that moment?* |  |  |  |  |  | b) Labelling:  ………………….. |
| 10 | ***This boy is being chased by a monster.*** | Large card 5 | | | | | a) Pointing: |
|  | a) *How is this boy feeling? Show me the picture that matches his face.* | A | B | C | D | **E** | ………………….. |
|  | b) *How does he feel at that moment?* |  |  |  |  |  | b) Labelling:  ………………….. |

## Emotion Comprehension Task Scoring Key

Sub-task 1 Sub-task 2

| Item 1 | 1 | Anger |  | Item 6 | 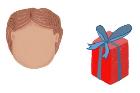1 | A | Sadness |
| --- | --- | --- | --- | --- | --- | --- | --- |
|  | 2 | Sadness |  |  |  | **B** | **Happiness** |
|  | 3 | Neutral |  |  |  | C | Neutral |
|  | **4** | **Happiness** |  |  |  | D | Fear |
|  | 5 | Fear |  |  |  | E | Anger |
| Item 2 | 6 | Anger |  | Item 7 | 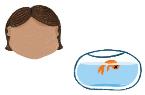2 | A | Anger |
|  | 7 | Neutral |  |  |  | B | Neutral |
|  | **8** | **Sadness** |  |  |  | C | Happiness |
|  | 9 | Fear |  |  |  | **D** | **Sadness** |
|  | 10 | Happiness |  |  |  | E | Fear |
| Item 3 | 11 | Fear |  | Item 8 | 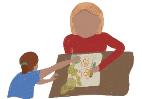 3 | A | Sadness |
|  | 12 | Happiness |  |  |  | **B** | **Anger** |
|  | 13 | Sadness |  |  |  | C | Happiness |
|  | **14** | **Anger** |  |  |  | D | Neutral |
|  | 15 | Neutral |  |  |  | E | Fear |
| Item 4 | 16 | Happiness |  | Item 9 | 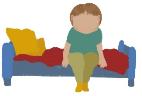4 | A | Fear |
|  | 17 | Fear |  |  |  | **B** | **Neutral** |
|  | 18 | Anger |  |  |  | C | Happiness |
|  | **19** | **Neutral** |  |  |  | D | Anger |
|  | 20 | Sadness |  |  |  | E | Sadness |
| Item 5 | 21 | Anger |  | Item 10 | 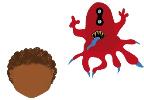5 | A | Happiness |
|  | **22** | **Fear** |  |  |  | B | Neutral |
|  | 23 | Sadness |  |  |  | C | Sadness |
|  | 24 | Happiness |  |  |  | D | Anger |
|  | 25 | Neutral |  |  |  | **E** | **Fear** |

| Locomotor activity: Scoring grid Please complete at least 5/9 skills (by checking the box corresponding to your observations) and answer yes (Y) or no (N) to each of the last 5 questions. | | Name : | | Name : | | Name : | | Name : | | Name : | | Name : | | Name : | | Name : | | Name : | | Name : | | Name : | | Name : | |
| --- | --- | --- | --- | --- | --- | --- | --- | --- | --- | --- | --- | --- | --- | --- | --- | --- | --- | --- | --- | --- | --- | --- | --- | --- | --- |
| ***Balancing*** | bench | 1 | | 1 | | 1 | | 1 | | 1 | | 1 | | 1 | | 1 | | 1 | | 1 | | 1 | | 1 | |
|  | beam | 2 | | 2 | | 2 | | 2 | | 2 | | 2 | | 2 | | 2 | | 2 | | 2 | | 2 | | 2 | |
| ***Climbing*** wall bars | with assistance | 1 | | 1 | | 1 | | 1 | | 1 | | 1 | | 1 | | 1 | | 1 | | 1 | | 1 | | 1 | |
|  | without assistance | 2 | | 2 | | 2 | | 2 | | 2 | | 2 | | 2 | | 2 | | 2 | | 2 | | 2 | | 2 | |
| ***Crawling***  over a 5m distance | using knees 3 or more times | 1 | | 1 | | 1 | | 1 | | 1 | | 1 | | 1 | | 1 | | 1 | | 1 | | 1 | | 1 | |
|  | using knees 1 or 2 times | 2 | | 2 | | 2 | | 2 | | 2 | | 2 | | 2 | | 2 | | 2 | | 2 | | 2 | | 2 | |
|  | without using knees | 3 | | 3 | | 3 | | 3 | | 3 | | 3 | | 3 | | 3 | | 3 | | 3 | | 3 | | 3 | |
| ***Crossing***  an obstacle in height | of 1m high | 1 | | 1 | | 1 | | 1 | | 1 | | 1 | | 1 | | 1 | | 1 | | 1 | | 1 | | 1 | |
|  | of 1m50 high | 2 | | 2 | | 2 | | 2 | | 2 | | 2 | | 2 | | 2 | | 2 | | 2 | | 2 | | 2 | |
|  | of 2m high | 3 | | 3 | | 3 | | 3 | | 3 | | 3 | | 3 | | 3 | | 3 | | 3 | | 3 | | 3 | |
| ***Crossing***  an obstacle in width | narrow passageway (30cm) | 1 | | 1 | | 1 | | 1 | | 1 | | 1 | | 1 | | 1 | | 1 | | 1 | | 1 | | 1 | |
|  | middle passageway (60cm) | 2 | | 2 | | 2 | | 2 | | 2 | | 2 | | 2 | | 2 | | 2 | | 2 | | 2 | | 2 | |
|  | wide passageway (90cm) | 3 | | 3 | | 3 | | 3 | | 3 | | 3 | | 3 | | 3 | | 3 | | 3 | | 3 | | 3 | |
| ***Hanging*** and ***swinging*** | hang with 2 hands | 1 | | 1 | | 1 | | 1 | | 1 | | 1 | | 1 | | 1 | | 1 | | 1 | | 1 | | 1 | |
|  | swing with 2 hands | 2 | | 2 | | 2 | | 2 | | 2 | | 2 | | 2 | | 2 | | 2 | | 2 | | 2 | | 2 | |
|  | by the feet | 3 | | 3 | | 3 | | 3 | | 3 | | 3 | | 3 | | 3 | | 3 | | 3 | | 3 | | 3 | |
| ***Jumping***  on the trampoline from a plot | lowest (step type) plot | 1 | | 1 | | 1 | | 1 | | 1 | | 1 | | 1 | | 1 | | 1 | | 1 | | 1 | | 1 | |
|  | medium (chair type) plot | 2 | | 2 | | 2 | | 2 | | 2 | | 2 | | 2 | | 2 | | 2 | | 2 | | 2 | | 2 | |
|  | highest (table type) | 3 | | 3 | | 3 | | 3 | | 3 | | 3 | | 3 | | 3 | | 3 | | 3 | | 3 | | 3 | |
| ***Sliding***  on an inclined plane | sitting | 1 | | 1 | | 1 | | 1 | | 1 | | 1 | | 1 | | 1 | | 1 | | 1 | | 1 | | 1 | |
|  | head forward | 2 | | 2 | | 2 | | 2 | | 2 | | 2 | | 2 | | 2 | | 2 | | 2 | | 2 | | 2 | |
|  | standing | 3 | | 3 | | 3 | | 3 | | 3 | | 3 | | 3 | | 3 | | 3 | | 3 | | 3 | | 3 | |
| ***Throwing***  a 3kg bag in hoops | close (40cm) | 1 | | 1 | | 1 | | 1 | | 1 | | 1 | | 1 | | 1 | | 1 | | 1 | | 1 | | 1 | |
|  | middle (80cm) | 2 | | 2 | | 2 | | 2 | | 2 | | 2 | | 2 | | 2 | | 2 | | 2 | | 2 | | 2 | |
|  | far away (120cm) | 3 | | 3 | | 3 | | 3 | | 3 | | 3 | | 3 | | 3 | | 3 | | 3 | | 3 | | 3 | |
| Globally, the child... | |  | |  | |  | |  | |  | |  | |  | |  | |  | |  | |  | |  | |
| (1) agreed to take the agility trail proposed | | Y | N | Y | N | Y | N | Y | N | Y | N | Y | N | Y | N | Y | N | Y | N | Y | N | Y | N | Y | N |
| (2) attempted to complete each obstacle (with or without assistance) | | Y | N | Y | N | Y | N | Y | N | Y | N | Y | N | Y | N | Y | N | Y | N | Y | N | Y | N | Y | N |
| (3) evolved his actions between the obstacles to better overcome them | | Y | N | Y | N | Y | N | Y | N | Y | N | Y | N | Y | N | Y | N | Y | N | Y | N | Y | N | Y | N |
| (4) dared to venture into acrobatic or unbalanced situations despite his fear | | Y | N | Y | N | Y | N | Y | N | Y | N | Y | N | Y | N | Y | N | Y | N | Y | N | Y | N | Y | N |
| (5) was satisfied with his performance on the trail and proud of his personal accomplishment | | Y | N | Y | N | Y | N | Y | N | Y | N | Y | N | Y | N | Y | N | Y | N | Y | N | Y | N | Y | N |
| Sum of points obtained (yes/no = 1/0 point) | |  | |  | |  | |  | |  | |  | |  | |  | |  | |  | |  | |  | |
| Maximum possible points | |  | |  | |  | |  | |  | |  | |  | |  | |  | |  | |  | |  | |
